# Complete ablation of tumor necrosis factor decreases the production of IgA, IgG, and IgM in experimental central nervous system tuberculosis

**DOI:** 10.22038/ijbms.2020.37947.9021

**Published:** 2020-05

**Authors:** Ngiambudulu M. Francisco, Nasiema Allie, Boipelo Sebesho, Bernhard Ryffel, Muazzam Jacobs

**Affiliations:** 1 Program of Infection and Immunity, the Fifth Affiliated Hospital of Sun Yat-sen University, Zhongshan School of Medicine, Sun Yat-sen University, Guangdong, China; 2 Institute of Tuberculosis Control, Zhongshan School of Medicine, Sun Yat-sen University, Guangzhou, China; 3 Key Laboratory of Tropical Diseases Control, Ministry of Education, Sun Yat-sen University, Guangzhou, China; 4 SAMRC Centre for Tuberculosis Research, Division of Molecular Biology and Human Genetics, Department of Biomedical Sciences, Stellenbosch University, Stellenbosch, South Africa; 5 Division of Immunology, Department of Pathology and Institute of Infectious Disease and Molecular Medicine, Faculty of Health Sciences, University of Cape Town, Cape Town 7925, South Africa; 6 Institut de Transgenose, CNRS, GEM2358, Orleans, France, University of Orleans and CNRS UMR7355, Experimental and Molecular Immunology and Neurogenetics, Orleans, France; 7 National Health Laboratory Service, Sandringham, Johannesburg, South Africa; 8 Immunology of Infectious Disease Research Unit, South African Medical Research Council, Cape Town 8000, South Africa

**Keywords:** Antibody, Central nervous system, Humoral, Immunity infections, Mycobacterium, Tumor necrosis factor

## Abstract

**Objective(s):**

This study aimed to explore the contribution of tumor necrosis factor (TNF) in the recruitment of B-cell and secretion of immunoglobulins (Igs) during cerebral tuberculosis (TB).

**Materials and Methods:**

In this work, the contributing role of TNF in regulating Ig secretions was investigated by comparing wild type TNF (TNF^f/f^), B-cell-derived TNF (BTNF^-/-^), and complete TNF ablation (TNF^-/-^) in a mouse cerebral *Mycobacterium tuberculosis* infection. Using flow cytometry and ELISA, we were able to examine the recruitment of B-cell subsets, and the production of Igs; also assessed the expression of surface markers on B cell subsets.

**Results:**

Here, we found that TNF^-/-^ mice showed defective expression of IgA, IgG, and IgM antibodies compared with TNF^f/f^ and BTNF^-/-^ mice, which was significantly decreased in the expression of surface markers and co-stimulatory molecules. Moreover, mice that produced low antibody levels were not able to control infection, therefore progressed to disease; providing direct evidence for the TNF gene-regulating humoral immunity during central nervous system (CNS) *M. tuberculosis* infection. In contrast, BTNF^-/-^ mice controlled the infection and had levels of IgA, IgG, and IgM comparable to TNF^f/f^ mice.

**Conclusion:**

Together, our results demonstrate that TNF may serve as an essential regulator of antibody-mediated immune responses in CNS TB. However, the protective level exhibited by TNF-producing B cells could be defined as baseline protection that could be used in the development of new therapeutic targets or designing new vaccines.

## Introduction

Tuberculosis (TB), a disease caused by a bacteria called *Mycobacterium tuberculosis *in humans, is currently the leading cause of death from infectious diseases globally despite being a treatable and curable disease (1). The recent global effort in the fight against TB is without a doubt being threatened with the emergence of drug resistance, in particular, rifampicin-resistant or multidrug-resistant (MDR) TB and extensively-drug-resistant (EDR) TB (2). The central nervous system (CNS) *M. tuberculosis* infection is a devastating condition, it causes a high level of mortality with a significant distressing level of cerebral morbidity; and in most cases with permanent neurological complications (3-5). CNS TB occurs when there is a structural rupture of subpial or subependymal foci, following lymphohematogenous dissemination of *Mycobacteria *from the primary lung infection (6), or rupture of the lung cavity (7). The immunological mechanism(s) underlying mycobacterial control *in vivo* is still not yet fully understood (8). The well-known crucial host-protective structure during TB is the formation of structures called granulomas in the affected organ. Granuloma is a condensed aggregate of immune and non-immune cells, such as epithelioid cells, dendritic cells, macrophages, neutrophils, fibroblasts, natural killer cells, B cells, and T cells (9, 10). In the brain, the above mentioned condensed cellular structure is referred to as rich foci. Yet, the role of several such cell-types in this compact structure is still unclear. 

It is widely accepted that these cell-types, including B lymphocytes, are recruited and regulated by proinflammatory cytokines including tumor necrosis factor (TNF). In fact, growing evidence implicates TNF in CNS TB (11-15). Recently, we showed the critical role of TNF in different cellular sources and organ-type such as lung (16) and brain (17, 18) in TB infection in a mouse model. The latest development in the biology of B cell has led to the subdivision of B cells into different cytokine-producing effector or regulatory subsets, which were detected *in vitro *and* in vivo* (19). Moreover, transcription of TNF is one of the initial events that occur after B lymphocytes stimulation via its antigen receptors, the induction of this transcription takes place within a half-hour of stimulation, and it does not necessitate *de novo* protein synthesis (20). It is now believed that B cells, as well as other lymphocytes, can obtain access into the neurological environment at different brain sites, such as the blood-brain barrier (BBB) (21, 22). CNS, which was previously considered as immune-privileged, has recently been reported to have lymphatic vessels (22), therefore, losing its immune privilege. These vessels may facilitate not only the maturation of immune cells but also provide a platform for the activation of infiltrating cells involved in cerebral immune responses. B cells regulate also the immune response, and they act as antigen-presenting cells (APC), but their immune regulatory functions are mainly restricted to follicular (Fo) B cells and plasmablasts because they share numerous properties with innate immune cells; thus they are also called innate-like B cells (ILBs) (23). In contrast, the B-cell subset that does not share properties with ILBs, but is capable of secreting optimal antibodies is classified as plasma cells. During TB, B cells are well recognized as the main player in adaptive immunity involving cellular response (24). While the cellular immune response is crucial, the role of humoral immunity during TB is still undefined (25, 26). Although no study has shown the efficacy of B-cell targeted vaccine and therapy in clinical trials, a clear understanding of the functional response and mechanism of antibody-producing-B-cell are important for the future design of TB vaccine and therapy. Despite the fact that B lymphocyte and antibody deficiencies may not be risk factors for human TB (27), mice with B cell ablation are susceptible to the infection (28-30), also non-human primates with B cells depletion had an increase in lesional bacterial burden (31). Yet, the mechanism(s) by which B cells influence tuberculous infection is still unclear, and the importance of TNF in the regulation of antibodies in health and TB remain an unexplored aspect. Hence, here, we aimed to define the role of TNF in the production of functional Ig antibody repertoire during CNS TB. Using a genetic approach, we demonstrate that complete ablation of TNF (TNF^-/-^) in mice resulted in an increased bacterial load and defective expression of antibody productions compared with control mice (TNF^f/f^) and B-cell-derived TNF (BTNF^-/-^) mice; which were associated with a significantly decreased expression of surface markers and co-stimulatory molecules. Together, these data underline the TNF gene as an immune regulator of antibodies in TB, also they provide mechanistic insights into the function of antibodies in controlling *M. tuberculosis* infection.

## Materials and Methods

***Mice***

All mouse strains, TNF-floxed (TNF^f/f^) as wild-type, B-cell-derived TNF deficiency (BTNF^-/-^) and complete TNF-deficient (TNF^-/-^) mice were previously studied (32). These animals were bred and maintained under specific pathogen-free conditions at the animal facility of the health science faculty, University of Cape Town (South Africa). Adult mice aged between 8 to 12 weeks, were PCR genotyped, thereafter infected mice were maintained under bio-safety level 3 conditions. 

***Intracerebral infection***

The *M. tuberculosis *laboratory strain*, *H37Rv, was grown in Middlebrook 7H9 broth containing 10% OADC and 0.5% Tween-80 until log phase at 37 ^°^C, thereafter aliquoted and directly stored at -80 ^°^C. Frozen aliquots were thawed, passed more than 30 times through a 29-gauge needle and diluted in sterile saline. The infection of animals was performed using a stereotaxic approach of directly injecting *M. tuberculosis *H37Rv into the cerebral cortex. Xylazine, 10 mg/kg Intervet (Zurich, Switzerland) and ketamine hydrochloride, 100 mg/kg (Bayer, Germany) were injected intraperitoneally in appropriate doses to anesthetize mice. Before the inoculation, we constructed a small burr hole anterior to the bregma and to the left of the midline in the skull, exposing the dura mater. Five mice per strain (unless otherwise stated) were inoculated intracerebrally with 1×10^4^ to 1×10^5^ colony forming units (CFUs) of *M. tuberculosis *H37Rv using a Hamilton syringe (Gas tight no. 1701, Switzerland). The burr hole was sealed with bone wax and the skin was sutured. All animals received a prophylactic painkiller for 3 days at 24 hr intervals. The clinical scoring system was adapted from previous reports (33, 34). Bodyweight of each mouse was scored daily for neurologic manifestations during the course of infection as follows: normal (no detectable signs)=0; head tilt=1; motility or decrease activity=2; behavior depression=3; and moribund state = 4. Organs of infected mice were harvested and processed at weeks 1, 2, 3, and 15 post-infection. Brain sections were hematoxylin and eosin (H&E)-stained, captured, and analyzed at week 3 after infection.

***Bacterial colony-forming unit enumeration***

Bacterial burdens in the spleens, lungs, and brains of mice (n=4 mice/group) infected with *M. tuberculosis* were determined at weeks 1, 2, and 3 after infection to monitor disease progression. Organs were weighed and homogenized in 0.04% Tween 80 saline. Thereafter, 10-fold serial dilutions of organ homogenates were plated in duplicates on Middlebrook 7H10 agar (Becton, Dickinson and Company) containing 10% OADC (Life Technologies, Gaithersburg, MD, USA), 0.5% glycerol and 25 µg/ml kanamycin. Mycobacterial cultures were incubated inside semi-sealed plastic bags at 37 ^°^C for 19 to 21 days. CFUs were calculated using the method previously described by Drennan *et al*. (35).

***Histology***

For histological studies, brain tissue samples were collected from TNF^f/f^, BTNF^-/-^, and TNF^-/- ^mice at week 3 after infection, and subsequently fixed in 10% normal buffered formalin, followed by paraffin embedment. Approximately 5-μm sections were stained with hematoxylin & eosin, captured and analyzed as previously described by Scott and Flynn (36).

***Quantitative real time-polymerase chain reaction***

Total RNAs were extracted and purified from brain tissues of mice infected for 3 weeks with *M. tuberculosis* using TRIzol reagent (Invitrogen, Carlsbad, CA, USA) according to the manufacturer’s instructions. Reverse transcription was then carried out by using 1 µg of RNA extracted. For quantitative real time-polymerase chain reaction (qRT-PCR), the reactions were performed in triplicate with each cDNA template by using IQ SYBR Green Supermix (Bio-Rad, Munich, Germany) and performed on Bio-Rad CFX96 real-time detection system (Bio-Rad). The expression for the following genes were determined CCR3 (5’-ACTTGGCAATTTCTGACCTGC-3’     and5’-AGGGCAAACACAGCATGGAC-3’),      CXCR4(5’-TCTTTGTCATCACACTCCCC-3’     and5’-CCTTTTCAGCCAGCAGTTTC-3’),      CCR5(5’-TCCTGCTCACACTACCATTC-3’     and5’-CCAAAGTTGACCGTTCTGAC-3’), CXCR5(5’-AGGAAAACGAAGCGGAAAC-3’     and5’-CCAGCAGAGGAAGAAAATGC-3’),      CCR6(5’-CAGTTACTCATGCCACCAAC-3’and5’-TGCCACACAGATGACCTTAC-3’),      CCR7(5’-TCAAGACCATGACGGATACC-3’and     5’-CGGTCAATGCTGATGCATAG-3’),      andβ-actin (5’-CGGTTCCGATGCCCTGAGGCTCTT-3’and 5’-CGTCACACTTCATGATGGAATTGA-3’) were used as sense and antisense primers. Expression levels of mRNAs were presented as fold change relative to wild-type mice, using the 2(-Delta Delta CT) method, and β-actin as the reference control as described previously (37). For the detection of nNOS, the following previously published primers: 5’-CTTCAAGAAGCTAGCAGAAGCTGT-3’ and 5’-ACAAGGACCAGAGTTTCATGTTC-3’ were used as sense and antisense primers, respectively (38).

***Flow cytometry analysis***

To analyze the surface marker expression on Fo B cell, plasmablast, and plasma cells, the brains of the mice were collected to generate single-cell suspensions. Nonspecific binding to cells was thereafter blocked through incubation with αFcγRIII (1 mg/ml of rat α-mouse CD32/16c); and the following antibodies for surface markers were used: CD1, CD93, and B220. The Fo B cells were gated and identified as CD1d^mid^CD93^-^B220^+^ cells, according to the method previously described (39). In order to identify the plasmablasts and plasma cells, we isolated single cells and labeled them with specific B-cell (CD1d, CD138, and B220) markers. Plasmablast and plasma cells were analyzed and identified as CD1d^high^CD138^-^B220^high ^cells and CD138^+^B220^low ^cells, respectively (40). The absolute counts of each population gated were calculated automatically with the following formula: Absolute Fo B cells or plasmablasts or plasma cells count=(number of target population events/number of bead events collected) x (percentage of positive total cells). In addition, the positive Fo B cells, plasmablast and plasma cells were stained for IgA, IgG, and IgM, as well as surface markers MHC II, CD40, and CD86. Samples were analyzed on a FACS LSRFortessa (BD) flowcytometer using Cell Quest software (BD).

***ELISA assay***

For *in vivo* antibodies measurement, frozen supernatants from brain homogenates were prepared for antibody measurement using ELISA at weeks 1, 2, and 3 subsequent to intracerebral *M. tuberculosis* infection. IgA, IgG, and IgM antibody concentrations were determined as described (41). Cytokine IL-1β, IL-12, TNF, and IFN-γ productions were measured in the brain’s frozen supernatants of TNF^f/f^, BTNF^-/-^, and TNF^-/- ^mice at week 3 after intracerebral *M. tuberculosis* infection. Measurements were done using commercially available ELISA reagents (R&D Systems, China) according to the manufacturer’s instructions. Versamax Microplate Reader (Molecular Devices, LLC, CA, USA) with SoftMax software was used to measure the absorbance of antibody and cytokine concentrations.

***Statistical analysis***

GraphPad Prism 6 software was used to perform statistical analyses. An unpaired Student’s *t*-test, or Two-way analysis of variance were performed when looking for groups or times differences, followed by Tukey’s *post hoc* test to determine significance, a *P*-value<0.05 was regarded significant, and data’s standard deviations are shown.

***Ethics statement***

This study was conducted in accordance with the recommendations of the South African National Standard and the National Institutes of Health guide, for the Care and Use of Laboratory Animals (NIH Publications No. 8023, revised 1978). Institutional Animal Utilization protocols were approved by the Animal Research Ethics Committee, Health Science Faculty, at the University of Cape Town.

## Results

***B cell-specific TNF-knockout mice are not susceptible to CNS TB and show a moderate bacterial burden and inflammatory response***

**Figure 1:**
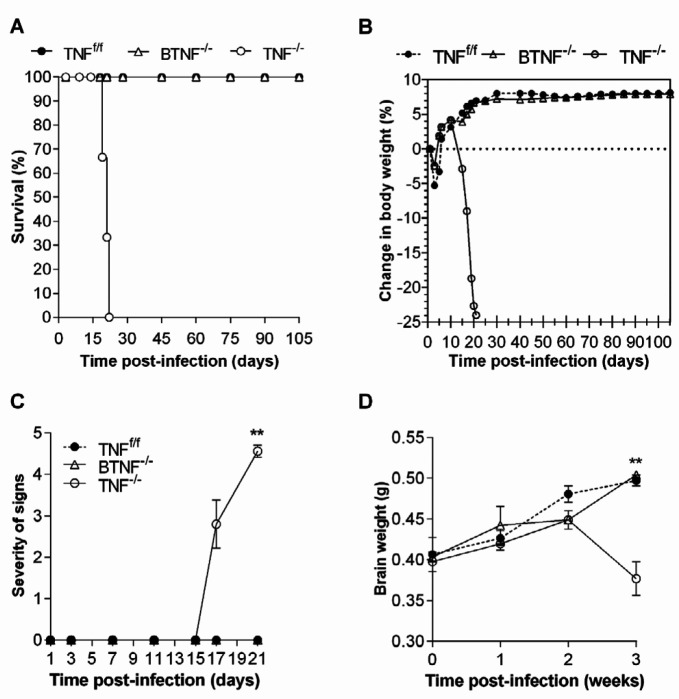
Disruption of tumor necrosis factor gene in B cells does not result in susceptibility in mice infected with *Mycobacterium tuberculosis*. TNF^f/f,^ BTNF^-/-^, and TNF^-/-^ mice were intracerebrally infected with *M. tuberculosis* at a dose of 1x10^4^ - 1x10^5^ CFU/brain, and 10 animals per group were used for (A) the percentage of survival and (B) change in bodyweight. (C) Severity of disease (recorded as clinical signs) and (D) brain weight were measured. Each experiment was repeated three or more times and representative data are shown as standard deviation (SD), **=*P<*0.01

Here, we disrupted the TNF gene in B cells to investigate the possible role of this gene during CNS TB, and compared with the complete TNF knockout mice and TNF^f/f^ mice. As expected, following *M. tuberculosis* infection, TNF^-/-^ mice succumbed to the infection just 3 weeks post-infection (Figure 1A), and dramatically lost bodyweight (Figure 1B), which was accompanied by severe clinical neurological signs (Figure 1C) and brain shrinking (Figure 1D) when compared with TNF^f/f ^mice. In contrast, mice with TNF deficiency in B cells controlled the infection similarly, compared with TNF^f/f ^mice (Figure 1). 

**Figure 2:**
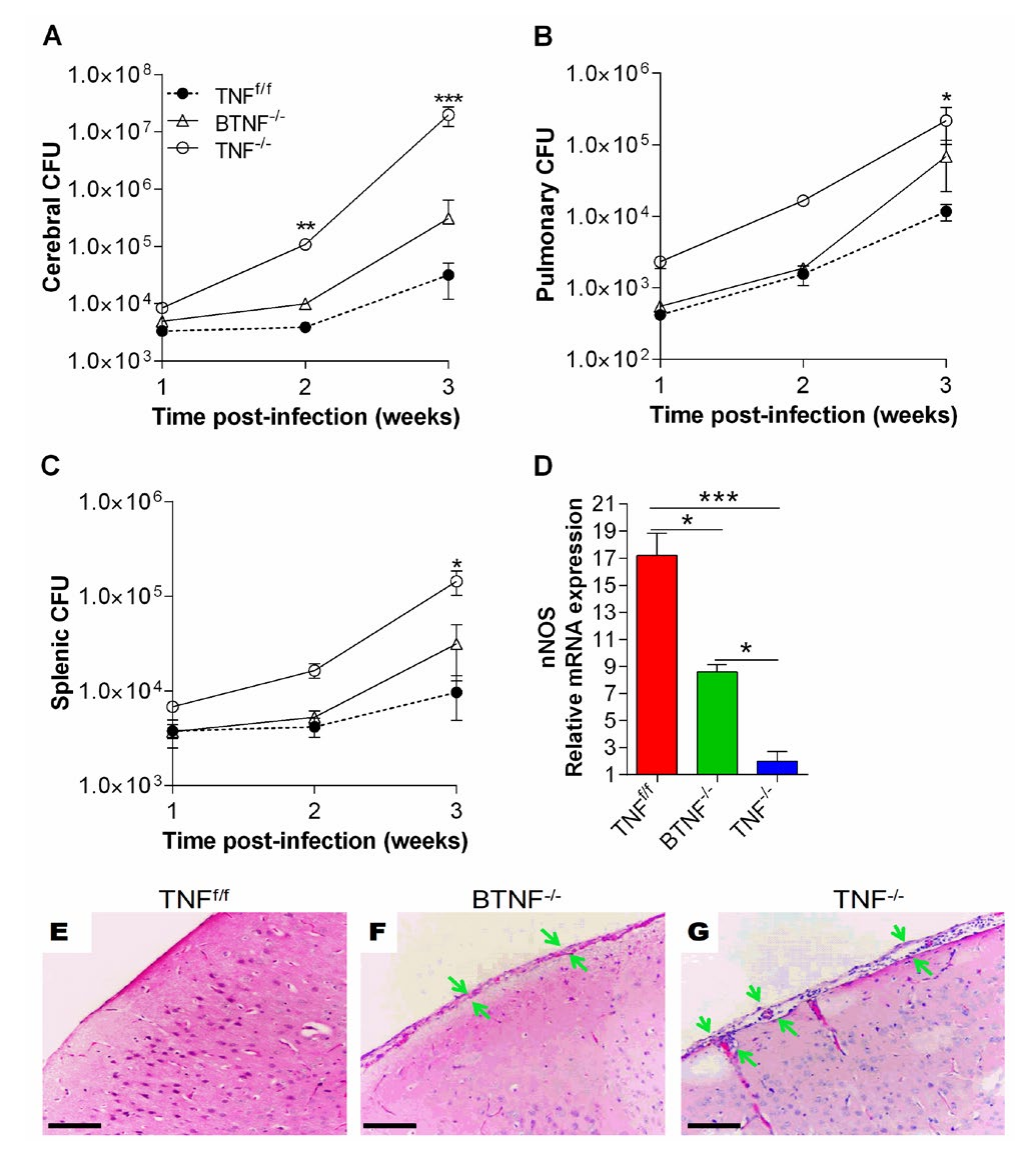
Tumor necrosis factor regulates dissemination of infection in complete TNF^-/-^ mice during acute central nervous system *Mycobacterium tuberculosis*. TNF^f/f^, BTNF^-/-^, and TNF^-/-^ mice were intracerebrally infected with *M. tuberculosis* at a dose of 1x10^4^ - 1x10^5^ CFU/mice. Bacterial loads of infected TNF^f/f^, BTNF^-/-^, and TNF^-/-^ mice (n=4 mice/group) were the number of viable bacteria present in the (A) brains, (B) lungs, and (C) spleens assessed at weeks 1, 2 and 3 post-infection. (D) nNOS mRNA expression in the infected brains of mice. mRNA was measured at day 21 post-infection. ΔCt=Ct (nNOS)–Ct (β-actin), Ct=cycle threshold, β-actin is the endogenous reference. (E-G) Hematoxylin-eosin–stained sections of the murine brain were performed in TNF^f/f^, BTNF^-/-^, and TNF^-/-^ mice intracerebrally infected with *M. tuberculosis* (1x10^5^ CFU). Each experiment was repeated three or more times and representative data are shown. *=*P<*0.05, ***=*P<*0.001 are regarded significant between TNF^f/f ^vs BTNF^-/-^ and TNF^-/-^ groups, green arrows indicate meningeal inflammation with leukocyte infiltration. Except for bacterial loads, at least 5 mice/group were used in all other experiments. Scale bar, 100 μm

We have previously shown that mice that completely lack TNF have increased cerebral *M. tuberculosis* bacterial burden and exacerbated pathology (17). Brains, lungs, and spleens of TNF^f/f^, BTNF^-/-^, and TNF^/- ^mice were collected for bacilli enumeration, and thereafter examined for the nNOS expression and the presence of infiltrating cells. As we previously reported (17), mice with complete loss of TNF gene showed increased mycobacterial burden in the brain, lung, and spleen when compared with TNF^f/f^ mice (Figures 2A–2C); also at week 3 post-infection, a striking increased bacterial burden was found in TNF^-/- ^mice compared with the B-TNF^-/-^ mice (Figure 2A). Moreover, in B cell-specific TNF-deficient mice, the bacterial burden was moderately increased, but no statistically significant difference was found in the brain at weeks 1 and 2 after infection, and in the lung and spleen when compared with the TNF^f/f ^mice or TNF^-/-^ mice at all time points (Figures 2A–2C). Next, we examined the implication of nNOS in the pathogenesis of CNS TB, brain tissues of intracranially *M. tuberculosis* infected TNF^f/f^, BTNF^-/-^, and TNF^-/- ^mice were used for nNOS mRNA expression. BTNF^-/- ^mice exhibited a significantly higher level of nNOS mRNA compared with TNF^-/-^ mice, but both mice groups showed significantly lower levels in comparison with TNF^f/f ^mice (Figure 2D). To gain insight into potential meningeal tissue damage caused by infiltrating immune cells, we stained brain tissues of infected TNF^f/f^, BTNF^-/-^, and TNF^-/- ^mice using hematoxylin and eosin staining. Brain tissues of BTNF^-/-^ mice showed mild meningeal leukocytic infiltration compared with the TNF^f/f^ mice (Figures 2E–2F). In contrast, mice with complete knockout of TNF showed similar degrees of infiltration and inflammation to what we previously have shown (17), with pronounced meningeal leukocytic infiltration (Figures 2E and 2G). In summary, our data suggested no role for TNF-divided B cells in host susceptibility to CNS TB, which could be explained by TNF compensation from other cellular sources such as microglia, neurons or astrocytes.

***Complete ablation of TNF modulates the infiltration of cerebral B-cell subsets in mice infected with M. tuberculosis***

**Figure 3:**
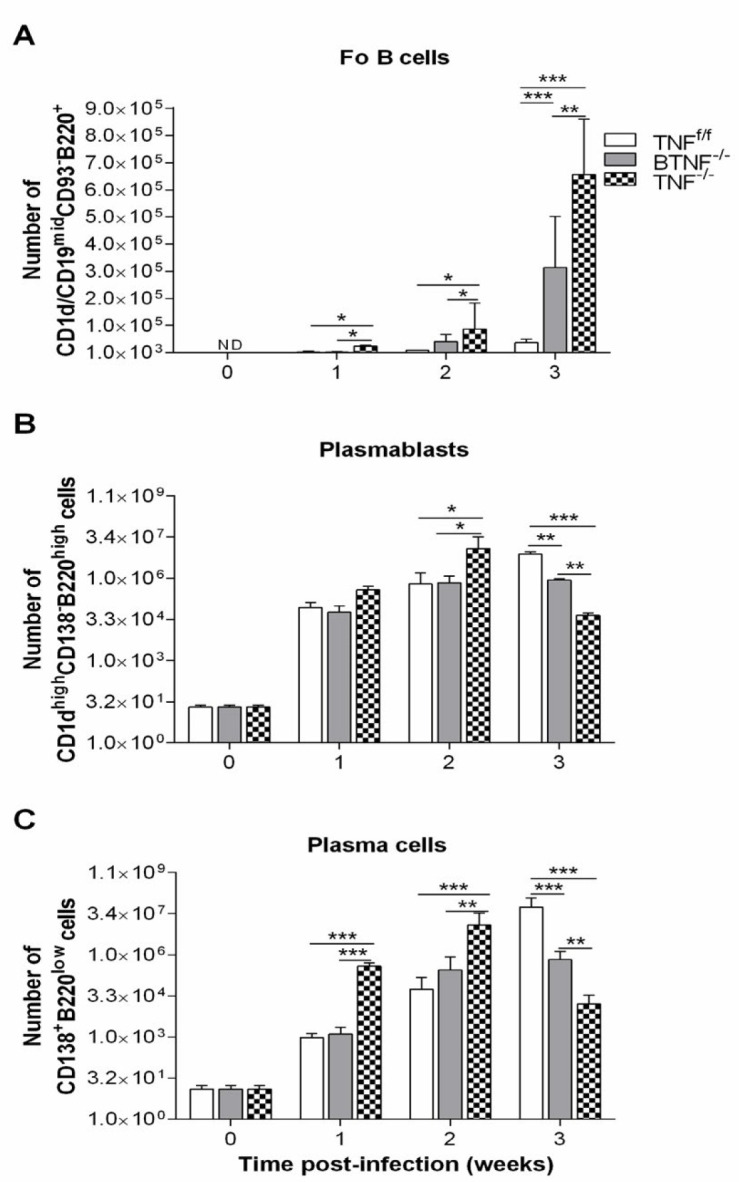
Loss of tumor necrosis factor function in mice led to increased Fo B cells and decreased plasmablast and plasma cell infiltrations after *Mycobacterium tuberculosis* infection. Flow cytometry analysis of infiltrating (A) Fo B cells, (B) plasmablasts, and (C) plasma cells population at different time points. Data are one representative of three independent experiments. Each experiment was repeated three or more times and yielded similar results, representative data are shown as SD of 5 mice/group. *=*P<*0.05, **=*P<*0.01, and ***=*P<*0.001

**Figure 4:**
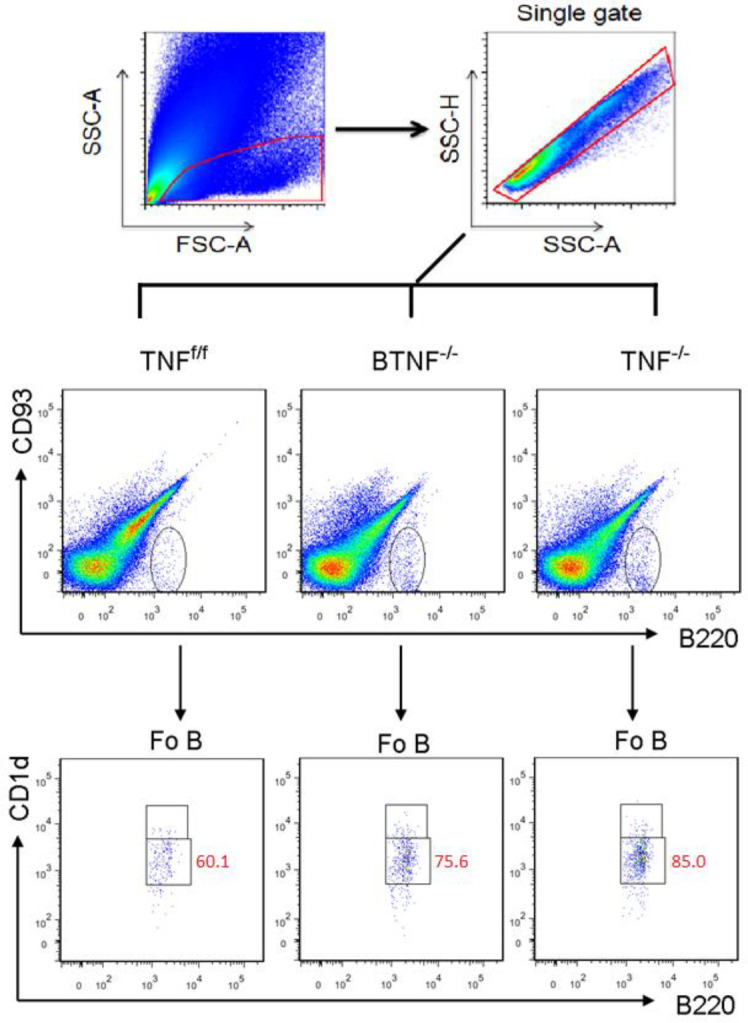
Loss of tumor necrosis factor function in mice led to increased Fo B cells and decreased plasmablast and plasma cell infiltrations after *Mycobacterium tuberculosis* infection. Flow cytometry analysis of infiltrating (A) Fo B cells, (B) plasmablasts, and (C) plasma cells population at different time points. Data are one representative of three independent experiments. Each experiment was repeated three or more times and yielded similar results, representative data are shown as SD of 5 mice/group. *=*P<*0.05, **=*P<*0.01, and ***=*P<*0.001

**Figure 5:**
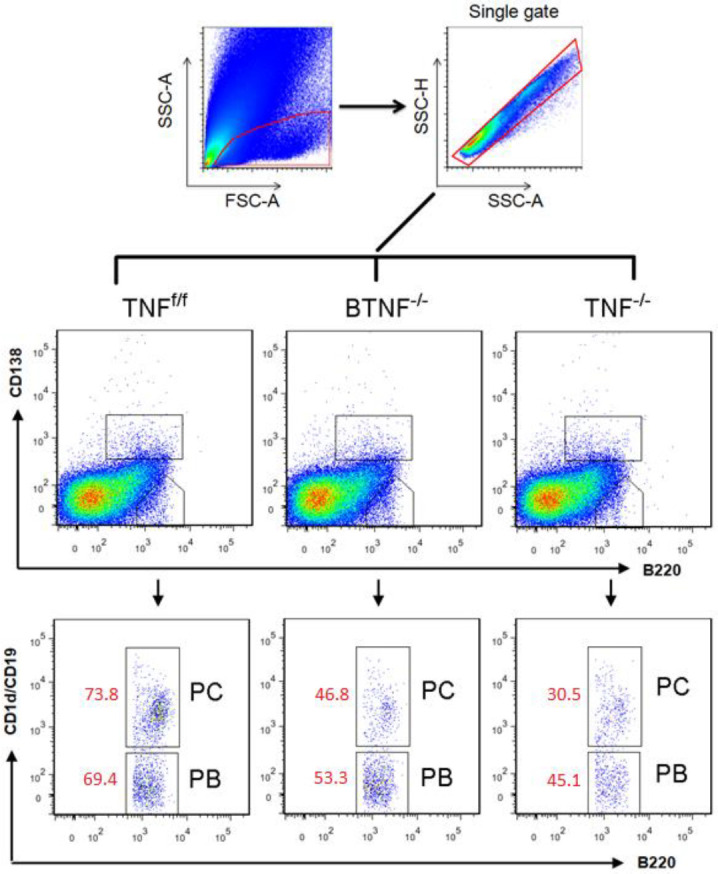
Tumor necrosis factor is necessary for adequate plasmablasts and plasma cells infiltration into the brain. Data shown are flow cytometry dot plots of plasmablasts (PB) and plasma cells (PC) population at week 3 post-infection. Isolated brain single cells from *Mycobacterium* *tuberculosis* infected TNF^f/f^, BTNF^-/-^, and TNF^-/-^ mice were labeled with CD1d, CD19, CD93, CD138, and B220 markers, and two B-cell subsets were identified as PB CD1d^high^CD138–B220^high^ and PC CD138+ B220^low^. This experiment was repeated three or more times, data are a pool of these repeats of 5 mice/group

To test whether complete ablation of TNF influences the infiltration of Fo B cells, plasmablasts, and plasma cells into the CNS during TB. Animals were intracerebrally infected, no significant difference was observed with regard to the absolute number of infiltrating Fo B cells in BTNF^-/-^ mice compared with TNF^f/f ^mice at weeks 1 and 2 after *M. tuberculosis* infection. Nevertheless, at week 3 after infection, the absolute numbers of infiltrating Fo B cells in BTNF^-/-^ mice were significantly (*P*<0.05) higher compared with TNF^f/f^ mice (Figure 3A and Figure 4). Additionally, the number of Fo B cells was significantly higher in the TNF^-/- ^from weeks 1, 2. and 3 after infection compared with TNF^f/f^ or BTNF^-/- ^mice (Figure 3A and Figure 4). Next, when we analyzed the infiltration of plasmablasts, no significant difference was detected in the absolute number of infiltrating plasmablasts in BTNF^-/-^ mice compared with TNF^f/f ^mice at weeks 1 and 2 post-infection. *M. tuberculosis*-infected BTNF^-/-^ mice presented significantly (*P*<0.05) lower absolute numbers of infiltrating plasmablasts in comparison with the control TNF^f/f^ mice at week 3 after infection (Figure 3B and Figure 5). In contrast, plasmablasts number was significantly higher in the TNF^-/- ^at week 2 after infection and thereafter significantly decreased at week 3 after infection when compared with TNF^f/f^ and BTNF^-/- ^mice (Figure 3B and Figure 5). The infiltrating number of cerebral plasma cells had no significant difference in BTNF^-/- ^mice at weeks 1 and 2 after infection compared with TNF^f/f^ mice, in contrast, a significant decrease was observed at week 3 post-infection when compared with TNF^f/f ^mice (Figure 3C and Figure 5). In the complete TNF knockout mice, we noticed striking higher infiltration of plasma cells at weeks 1 and 2 post-infection compared with TNF^f/f ^or BTNF^-/-^ mice (Figure 3C and Figure 5). Moreover, at week 3 post-infection, the number of infiltrating plasma cells significantly decreased when compared with TNF^f/f ^or BTNF^-/-^ mice (Figure 3C and Figure 5). Together, the data indicated that complete TNF ablation is critical in the regulation of B-cell subset influx into the CNS.

***TNF deficiency mice exhibited a striking increase expression of chemokine receptors, but modulated the production of T helper 1 cytokines***

**Figure 6:**
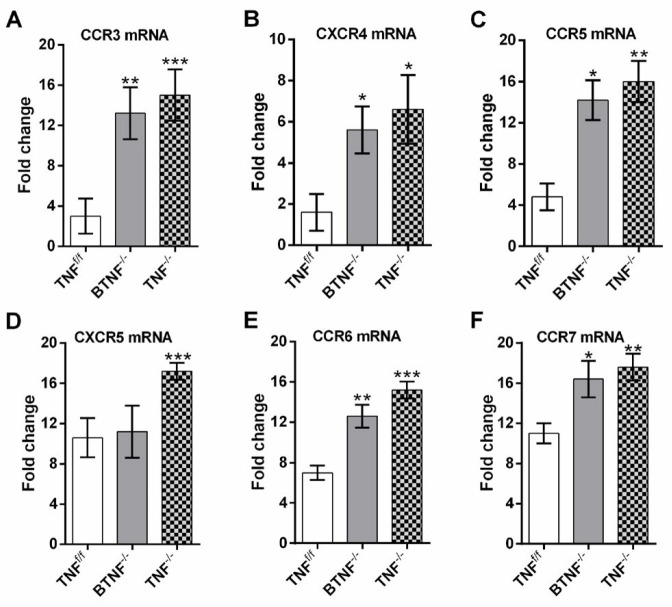
Tumor necrosis factor modulates the expression of chemokine receptors. Brain tissues (A) CCR3, (B) CXCR4, (C) CCR5, (D) CXCR5, (E) CCR6, and (F) CCR7 expressions in TNF^f/f^, BTNF^-/-^, and TNF^-/-^ mice infected with 1x10^5^ CFU/brain of *Mycobacterium tuberculosis*. Fold changes are shown as relative to control of wild-type mice (TNF^f/f^). The data represent a pool of three or more independent experiments and are shown as SD of 5 mice/group. *=*P<*0.05, **=*P<*0.01, ***=*P<*0.001

To investigate whether the infiltrated B-cell subsets received signal from the periphery, brain tissues from mice were collected at week 3 post-infection and following mRNA extraction. Chemokine receptors, including CCR3 (42), CXCR4 (43), CCR5 (44), CXCR5 (45), CCR6 (46), and CCR7 (47), which have been reported to be implicated in B cells chemotaxis in other disease models were quantified by qRT-PCR. The results reveal CCR3, CXCR4, CCR5, CCR6, and CCR7 were significantly expressed in mice with TNF deficiency (BTNF^-/- ^and TNF^-/-^ mice) compared with TNF^f/f ^mice (Figures 6A, B, C, E, F). Nevertheless, fold change relative to wild-type mice on CXCR5 was only significantly increased in TNF^-/-^ mice, but no statistical difference was found when compared with the BTNF^-/-^ group (Figure 6D). 

We also evaluated the global levels of cytokines IL-1β, IL-12, TNF, and IFN-γ in the brain’s supernatants of TNF^f/f^, BTNF^-/-^, and TNF^-/- ^mice. IL-1β, IL-12, and TNF levels in the TNF^-/- ^mice showed significant decreases compared with those of the controls TNF^f/f^ and BTNF^-/-^ mice at 3 weeks after infection (supplementary Figures 1A, B, C). The level of IFN-γ was significantly increased in TNF^-/- ^mice compared with those in the TNF^f/f^ and BTNF^-/-^ mice (supplementary Figure 1D). No significant difference was observed between BTNF^-/- ^versus TNF^f/f^ mice (supplementary Figures 1A, B, C, D). Unlike the complete deletion of the TNF gene, these results indicated that the deletion of TNF in B lymphocytes does not alter the Th1 immune response in CNS TB. 

***TNF is required for the regulation of immunoglobulins in CNS*** ***TB***

**Figure 7:**
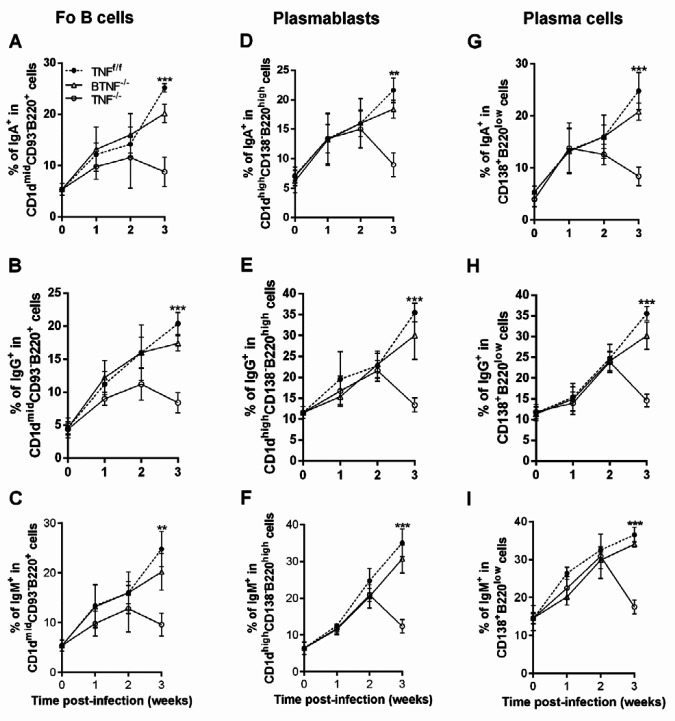
Tumor necrosis factor is critical for the regulation of immunoglobulin secretions in Fo B cells, plasmablasts and plasma cells in mice infected with *Mycobacterium tuberculosis*. (A, B, and C) IgA, IgG, and IgM expression in Fo B cells, (D, E, and F) IgA, IgG, and IgM expression in plasmablasts, and (G, H, and I) IgA, IgG, and IgM expressions in plasma cells of TNF^f/f^, BTNF^-/-^, and TNF^-/-^ mice. Data are presented as mean±SD of 5 mice/group. This experiment was repeated three or more times and representative data are shown as SD. *=*P<*0.05, **=*P<*0.01, ***=*P<*0.001

Next, we asked whether TNF was involved in impairing the secretion of antibodies in the infiltrating B-cell subsets. To investigate this, we analyzed the production of IgA, IgG, and IgM on Fo B cells, plasmablasts, and plasma cells in TNF^f/f^, BTNF^-/-^, and TNF^-/- ^mice at weeks 1, 2, and 3 after intracranial infection. Following infection, delayed and decreased recruitment of Fo B cell-producing IgA^+^, IgG^+^, and IgM^+^ were observed at week 3 after infection in the TNF^-/- ^mice compared with the TNF^f/f^ and BTNF^-/-^ mice (Figures 7A–7C). Brain Fo B cells of TNF^-/-^ mice had alterations in the expression of IgA, IgG, and IgM compared with TNF^f/f^ and BTNF^-/-^ mice at week 3 after intracranial infection (supplementary Figure 2). Thereafter, we examined the presence of plasmablast-producing IgA^+^, IgG^+^, and IgM^+^ in the TNF^f/f^, BTNF^-/-^, and TNF^-/- ^mice. We found that plasmablast-producing IgA^+^, IgG^+^, and IgM^+^ cells were similar at weeks 1 and 2 post-infection in all groups. Mice with complete loss of function of TNF failed to keep intact the recruitment of plasmablast-producing IgA^+^, IgG^+^, and IgM^+^ at week 3 post-infection, leading to a significant decrease of these cells when compared with TNF^f/f^ and BTNF^-/-^ mice (Figures 7D–7F and supplementary Figure 2). Finally, we assessed whether TNF is required for the recruitment of plasma cell-producing IgA^+^-, IgG^+^-, and IgM^+^-cells. There were no significant differences in weeks 1 and 2 after infection in all groups. In addition, mice with complete TNF ablation exhibited a marked decrease in the percentage of plasma cell-producing IgA^+^, IgG^+^, and IgM^+^ compared with TNF^f/f^ and BTNF^-/-^ mice (Figures 7G–7I and supplementary Figure 2). 

**Figure 8:**
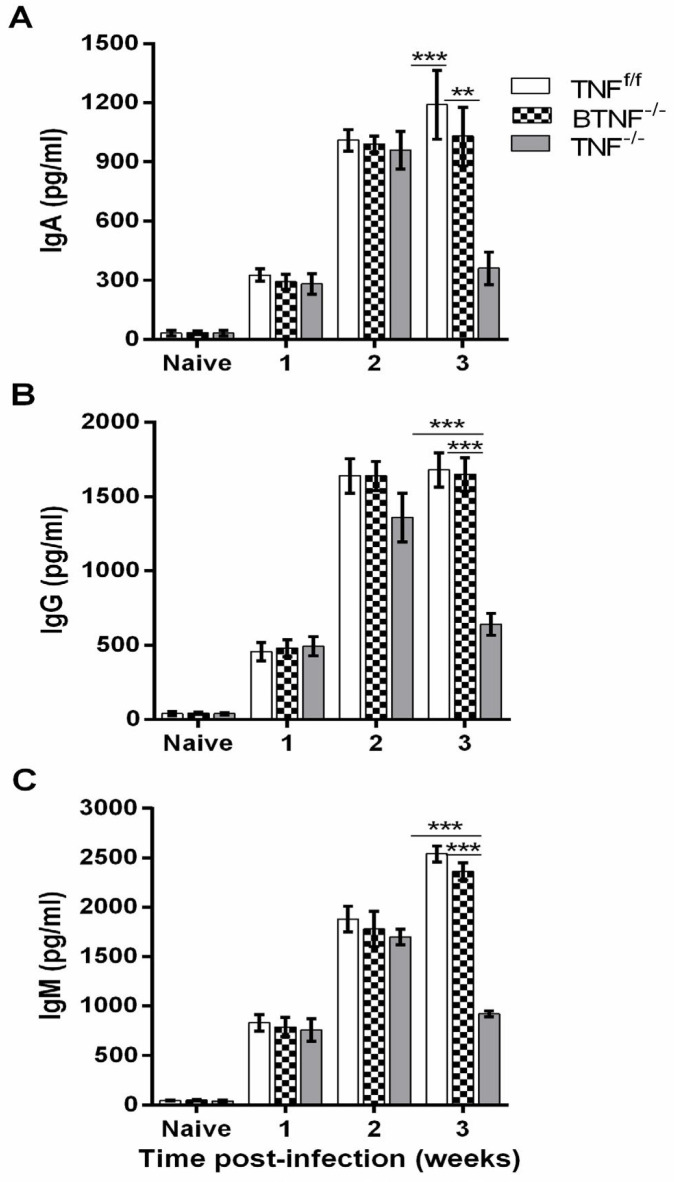
Decreased antibody productions in TNF^-/-^ mice infected with *Mycobacterium tuberculosis*. (A) IgA, (B) IgG, and (C) IgM antibody productions were measured from the supernatants of infected brains of TNF^f/f^, BTNF^-/-^, and TNF^-/-^ mice using ELISA (5 mice/group). Data represent a pool of two independent experiments shown as SD. Statistical analysis was performed defining differences to TNF^f/f^ and BTNF^-/-^ mice vs TNF^-/-^ mice. **=*P<*0.01, ***=*P<*0.001

We also tested the production of IgA, IgG, and IgM in naive groups of TNF^f/f^, BTNF^-/-^, and TNF^-/-^. Results show no significant difference with regard to IgA, IgG, and IgM production in the brain of naive mice (Figures 8A, B, and C). Similarly, no significant difference was observed at weeks 1 and 2 after infection. In contrast, strikingly reduced IgA, IgG, and IgM levels were found at week 3 after infection in mice with complete TNF ablation when compared with TNF^f/f^ and BTNF^-/-^ mice (Figures 8A, B, and C). Collectively, the data suggested that global deletion of TNF is critical, but TNF-producing B cell is not critical in regulating the recruitment of antibody-producing cells.

***Complete deletion of TNF impairs surface markers expression on B-cell subsets after M. tuberculosis infection***

**Figure 9.**
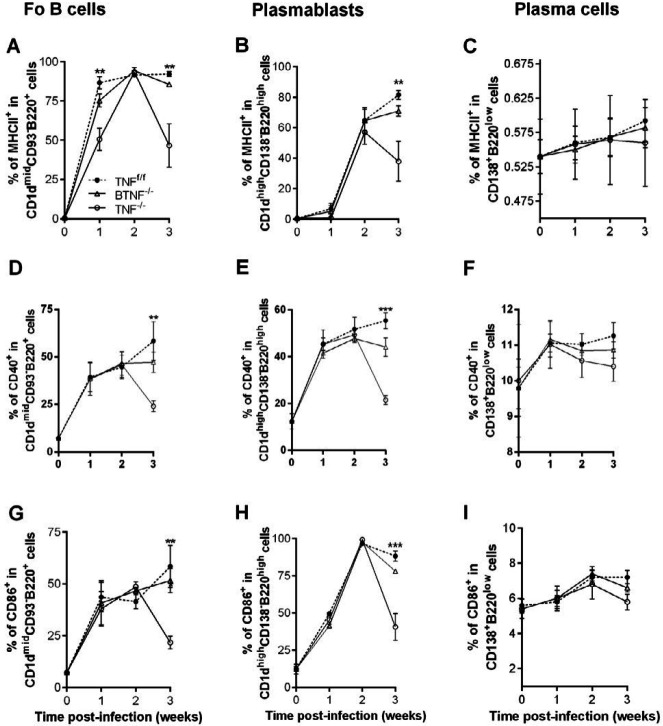
Complete ablation of tumor necrosis factor impairs the expression of surface markers in B-cell subsets during experimental central nervous system tuberculosis. Surface markers MHC II, CD40, and CD86 expressions in (A, D, and G) Fo B cells, (B, E, and H) plasmablasts, and (C, F, and I) plasma cells of TNF^f/f^, BTNF^-/-^, and TNF^-/-^ mice (5 mice/group). This experiment was repeated three or more times, data are a pool of these repeats and presented as SD. *=*P<*0.05, **=*P<*0.01, ***=*P<*0.001

To investigate whether TNF is involved in the expression of surface markers on B cells, we analyzed the expression of MHC II, CD40, and CD86 molecules as activation markers (48). We found that the percentage of infiltrating Fo B cell-expressing MHC II (Figure 9A) in TNF^-/-^ mice was delayed and significantly lower as early as week 1 after infection in BTNF^-/-^ and TNF^-/-^ mice compared with the TNF^f/f^ mice; moreover, no significant difference was seen at week 2 post-infection in any mouse strains. The expression and mean fluorescence intensity (MFI) of MHC II on brain Fo B cells of TNF^-/-^ mice were found to be significantly lower at week 3 post-infection compared with BTNF^-/-^ and TNF^f/f^ mice (Figure 9A; supplementary Figure 3 and supplementary Figure 4A). The expression levels of MHC II on plasmablasts showed no significant difference at week 1 and 2 after infection in any groups; on the contrary, the expression and MFI of MHC II on plasmablasts were significantly lower in TNF^-/-^ mice at week 3 after infection compared with TNF^f/f ^mice (Figure 9B; supplementary Figure 3 and supplementary Figure 4B). In contrast, no significant difference was noticed with regard to the expression and MFI of MHC II on plasma cells, which were almost not detectable in any animals at any time points (Figure 9C; supplementary Figure 3 and supplementary Figure 4C); this could possibly be due to its phenotyping characteristic as previously demonstrated (49). Finally, we examined the expression of CD40 and CD86 co-stimulatory markers. Like MHC II, we found no significant difference in the expression of CD40 and CD86 at weeks 1 and 2 after infection in any mice strains, moreover, a significantly lower expression and MFI of Fo B cell-expressing CD40 and CD86 (Figures 9D, G; supplementary Figure 3 and supplementary Figures 4D, G); and plasmablast-expressing CD40 and CD86 (Figure 9E, H; supplementary Figure 3 and supplementary Figures 4E, H) were found in TNF^-/- ^mice compared with TNF^f/f ^mice at week 3 after infection. As we expected, the expression and MFI of plasma cell-expressing CD40 and CD86 in BTNF^-/-^ and TNF^-/- ^mice were comparable to those of TNF^f/f ^mice (Figures 9F, I; supplementary Figure 3 and supplementary Figures 4F, I) at week 3 after infection. In contrast, when complete TNF loss occurs, Fo B cells infiltration is increased; suggesting that regulation of Fo B cells recruitment into CNS is absolutely dependent on the TNF gene.

## Discussion

In the presence of stimuli or pathogens, B cells respond by inducing antibodies and specific inflammatory mediators such as TNF. Regulation of the TNF gene is one of the first events that take place after stimulation of B cells (20). For this reason, we hypothesized that TNF may play a significant contributory role in regulating the CNS humoral immune response during *M. tuberculosis* infection. We, therefore, examined the overall contribution role of TNF and particularly the involvement of TNF produced by B lymphocytes in host-mediated humoral immune response directed against cerebral TB. Data presented here showed that global TNF is crucial in regulating humoral-immune-mediated cerebral TB pathology. Also, we showed for the first time that TNF is an absolute prerequisite in the regulation and production of antibodies during CNS-TB; we found in the mouse model, B-cell-derived TNF to be redundant. An optimal TNF level in dynamic homeostasis is important in controlling effectively the *M. tuberculosis* infection, however, a change in concentration level that favors either reduced or excessive levels has been shown to be detrimental to the host (15) and promotes the provision for mycobacterial persistence. The presence of infiltrating cerebral Fo B cells, plasmablasts, and plasma cells shown in this study indicate that these B-cell subsets, after their chemokine receptors receive signal, can migrate to the CNS during TB, and play a crucial role in regulating the host’s immunity. The increased level of infiltrating Fo B cells shown in TNF^-/-^ mice at week 3 after infection correlated with disease progression in this mouse strain, suggesting that uncontrolled infiltration of this B-cell subset is pathogenic, which underscore TNF requirement in controlling the infiltration of Fo B cells in CNS TB. Nevertheless, this observation could also be explained by the fact that this B-cell subset may play an APC-like role, by exercising cellular immunity than humoral immunity, as similarly increased infiltrations were previously reported in cells of the myeloid lineage such as neutrophils, macrophage, and dendritic cells in TNF^-/-^ mice at week 3 post-infection (17, 18).

A previous study using B-cell knockout mice showed the role for B lymphocytes in early immune responses during pulmonary *M. tuberculosis* infection, which was found to be associated with the development of lung pathology, but also delay in the *M. tuberculosis* dissemination, as well as alteration of cellular infiltrate in the lungs (50). Because the use of cell-specific gene-deficient mice has generated insight into regulatory roles of cells that express TNF (16-18, 32), in our study, we compared BTNF^-/- ^and TNF^-/-^ mice with the control wild type (TNF^f/f^). 

As expected, mice with complete TNF ablation succumbed to the infection as previously shown (17, 18), but BTNF^-/- ^mice in this study exhibited moderate bacterial burden and inflammatory response compared with TNF^f/f^ mice. When we assessed the infiltration of B cell-type, we observed that Fo B cells were significantly increased in BTNF^-/-^ and complete TNF knockout mice. The numbers of infiltrating plasmablast and plasma cells were decreased in the BTNF^-/-^ and TNF^-/-^ mice, yet chemokine receptor CCR3, CXCR4, CCR5, CXCR5, CCR6, and CCR7 were highly expressed in the BTNF^-/-^ and TNF^-/-^ mice compared with TNF^f/f^ mice. Nevertheless, we were unable to confirm whether the infiltrating cerebral B-cell subsets matured inside the brain, but it is worthwhile to mention that the occurrence of lymphoid follicle-like structures in the meninges of the brain has previously been reported in other CNS disease models, indicating that B-cell maturation can be sustained locally within the CNS, and greatly contribute to the establishment of a compartment that maintains humoral immunity (51, 52). The expression of MHC II, CD40, and CD86 in Fo B cells and plasmablasts was significantly decreased in TNF^-/-^ mice at week 3 after infection. The decreased percentages and MFI observed on MHC II-, CD40-, and CD86-expressing cells were associated with decreased Ig-expressing cells. While in plasma cells, the expression of MHC II, CD40, and CD86 showed no significant difference in any mouse strains. Previous studies reported that mature B cells express weak but detectable levels of MHC II, CD40, and CD86 (53, 54). Therefore, our data are in agreement with the previously reported phenotype of plasma cells. Data implicate TNF in the deregulation of surface markers produced on B-cell subsets and consequently predisposed the host to a very susceptible phenotype. 

Furthermore, in BTNF^-/- ^mice, we observed high percentage levels of antibodies resembling that of TNF^f/f ^mice. In contrast, the antibody secretions in B-cell subsets in TNF^-/-^ mice were significantly decreased. We speculated that the optimal humoral response found in BTNF^-/-^ mice could be due to the compensation of the TNF gene from other immune cells, including macrophage/microglia, dendritic cells and neurons, or the interaction of B cell with CD4^+ ^T cells, which triggers cytokine synthesis that reciprocally regulates the antibody responses of B cells (29, 55, 56). Amongst the cytokines produced by B lymphocytes, TNF has been shown to play an important role in B cell activation (57). Herein, we observed that the deletion of TNF in B-cells enhances their resistance by regulating cytokine production, but complete TNF ablation modulates cytokine response, which renders the host highly susceptible during CNS TB (17, 18), and ultimately leading to an inefficient humoral response in a murine model. In other disease models, studies have shown that TNF^−/−^ mice fail to produce an optimal B cell response (32, 58, 59). Future studies should investigate the role other cytokines produced by the recruited B cells play in CNS TB. Previous studies focusing on humoral immunity support antibody as the mediator of the immune response, whereas those studying cellular immunity championed phagocytic cells as key mediators of host immune defense, with antibody being a backup constituent primarily to promote opsonization.

Host immune cells can contribute to the TNF global level required for optimum control of mycobacteria in different ways. One of them is the compensatory response of TNF from different cellular sources. Studies *in vitro* and those involving animals, non-human primate, and human models showed that susceptibility to mycobacterial infection can result from either excessive or inadequate TNF levels (13, 15, 24, 60-62). Under excessive TNF conditions, it promotes macrophage/microglia necrosis, which enriches conditions for uncontrolled bacilli growth. In inadequate conditions, the effector function of immune cells is impaired, and this functional impairment leads to intracellular *M. tuberculosis* replication and eventually cell lysis. Here, we show that cellular- and humoral-mediated immune responses are both regulated during CNS TB; and it is obvious that they may function synergistically. This study and those from other groups (29, 63-67) challenge the common belief that the immune response against *M. tuberculosis* relies solely on cellular defense mechanisms, which relegates the role of B cells in TB humoral immunity. Using TNF deficient mice, we cleared the inconsistent dogma by showing that even the B-cell subset that does not take part in cellular immunity has decreased the level of the humoral response. Therefore, the progression to a disease state with a chronic inflammatory response and high bacterial burden could result in the production of less effective antibodies as immunity to disease evolves. But importantly, the functional differences of these antibodies also reflect differences in specificity to disease progression. A hallmark study by Lu *et al*. (68) showed the existence of distinct antibody Fc effector profiles that correlate with different TB disease states. Their results also showed that antibodies in individual with latent TB infection (LTBI) have enhanced Fc effector. In addition, antibodies in their LTBI subjects enhanced several macrophage responses against intracellular *M. tuberculosis*, including inflammasome activation independent of pyroptosis and phagolysosomal maturation (68), but in their study, they unraveled the molecule(s) involved in the mechanism underlying humoral immunity that drives pathogenesis.

Here, we show that BTNF^-/-^ mice, which did not progress to a disease state and behaved like in LTBI state, controlled the infection by producing a better level of antibodies that correlated protection. It is worthwhile emphasizing here that the protective TNF concentration observed in BTNF^-/-^ mice could be the result of TNF compensatory from other cellular sources. Therefore, the functionally superior antibodies reported in our BTNF^-/-^ mice support the notion that those which make better antibodies are capable of controlling infection and do not progress to disease. However, we were unable to show whether *M. tuberculosis* infects or internalizes in B cells. While our study does not attempt to show the infectivity of *M. tuberculosis* in B cells, it provides a framework of key functional characteristics on which future research can be based. Our findings support the view that, during TB, both immune arms contribute to host defense. The data presented here suggest that TNF is critical in mediating humoral immunity during cerebral TB, and mice that made better antibodies were able to control cerebral *M. tuberculosis* infection and did not progress to disease. 

## Conclusion

We successfully showed that global TNF production is required in the production of antibodies. We also showed that complete ablation of TNF exacerbated the infiltration of B cells subsets into the brain. Finally, we showed that TNF-producing B cells is more likely to be protective. However, the protective level exhibited by TNF-producing B cells could be defined as baseline protection that could be used in the development of new therapeutic targets or designing new vaccines. Given the increased Fo B cell influx observed in this study, future studies should elucidate the pathogenic role that this B cell-subset may play in TB; and to investigate also the way in which TNF regulates the antibody sections during TB or other cerebral diseases. Our findings add to the ongoing discussion of the role of TNF and context-dependent function of B cells during TB, for which there is little appreciation.

## Data Availability

All data generated during this study are included in this manuscript and its supplementary information file.
